# Wind Energy Conversion by Plant-Inspired Designs

**DOI:** 10.1371/journal.pone.0170022

**Published:** 2017-01-13

**Authors:** Michael A. McCloskey, Curtis L. Mosher, Eric R. Henderson

**Affiliations:** 1 Department of GDCB, Iowa State University, Ames, IA, United States of America; 2 Creodyne, LLC, 2410 NE 13^th^ Ct, Ankeny, IA, United States of America; CNRS, FRANCE

## Abstract

In 2008 the U.S. Department of Energy set a target of 20% wind energy by 2030. To date, induction-based turbines form the mainstay of this effort, but turbines are noisy, perceived as unattractive, a potential hazard to bats and birds, and their height hampers deployment in residential settings. Several groups have proposed that artificial plants containing piezoelectric elements may harvest wind energy sufficient to contribute to a carbon-neutral energy economy. Here we measured energy conversion by cottonwood-inspired piezoelectric leaves, and by a “vertical flapping stalk”—the most efficient piezo-leaf previously reported. We emulated cottonwood for its unusually ordered, periodic flutter, properties conducive to piezo excitation. Integrated over 0°–90° (azimuthal) of incident airflow, cottonwood mimics outperformed the vertical flapping stalk, but they produced << daW per conceptualized tree. In contrast, a modest-sized cottonwood tree may dissipate ~ 80 W via leaf motion alone. A major limitation of piezo-transduction is charge generation, which scales with capacitance (area). We thus tested a rudimentary, cattail-inspired leaf with stacked elements wired in parallel. Power increased systematically with capacitance as expected, but extrapolation to acre-sized assemblages predicts << daW. Although our results suggest that present piezoelectric materials will not harvest mid-range power from botanic mimics of convenient size, recent developments in electrostriction and triboelectric systems may offer more fertile ground to further explore this concept.

## Introduction

The concept of wind energy harvesting by artificial plants has been considered in primary literature, patents, grants, and by commercial concerns [1–6]. In these schemes electromechanical coupling is achieved by piezoelectric elements assembled into the faux plant. If modest-sized piezoelectric plants could generate tens of watts (daW) off-grid, charging appliance batteries, they might supplement energy from residential turbines and commercial wind farms, helping to achieve the goal of “20% Wind Energy by 2030” [7]. Here we characterize a novel cottonwood-based piezo-leaf, and we predict size constraints on daW-scale harvesting by synthetic plants.

The rationale underlying fabrication of wind-harvesting plants is multifold. Visual and acoustic impacts of wind turbine farms are widely-shared concerns [1], and artificial plants may be less of a hazard to bats and birds than are wind turbines [8]. In the United States, geographic hotspots of average wind speed and population density are widely separated, and given limitations of the grid, local off-grid harvesting for reduced-scale application suggests a niche for piezo-plants. In previous efforts, Oh et al. (2009) built a miniature wind energy-harvesting tree of ten plastic leaves bearing polyvinylidene fluoride (PVDF) inserts that generated 47 mV peak voltage [3], and Zhang et al. (2014) fashioned a “leaf generator” from PZT nanofibers that produced 820 mV peak voltage in wind of 17 m/s [4]. Li et al (2011) developed a far more effective piezo-leaf with a “vertical flapping stalk” that generated ~ 100 to 300 μW in modest wind [5], and “for practical application” suggested assembly of devices with “hundreds or thousands of the Piezo-Leaves, like ivy, tree and forest.” Solar Botanic plans to tap both wind and solar energy from groves of “Energy Trees” sprouting their patented “Nanoleaf” [2]. The VTT Technical Research Center in Finland took this concept a step further by 3d printing of solar energy-harvesting leaves “capable of harvesting kinetic energy from a surrounding environment such as wind” [6].

The patent motion of leaves and branches in wind dissipates wind energy. Given a suitable transduction scheme, mimicry of plant anatomies could yield visually appealing architectures for mid-scale wind energy harvesting. Wind energy that is partitioned among leaves, branches, bole and roots of a tree induces motion at sub-Hz to a few Hz (Results), and in a 10 mph breeze a cottonwood tree with 500,000 leaves may dissipate 80 W via leaf motion alone (Appendix A in S1 File). If a faux tree scavenged half this power, it could deliver off-grid energy sufficient to charge batteries of small household appliances. Tree leaves in the genus *Populus* flutter in wind speeds at or below the ~ 7 mph threshold of conventional turbines [9], suggesting deployment of faux trees in populous settings where tall, noisy turbines are viewed a nuisance. Cell phone towers camouflaged as trees already have been used for decades in some urban settings, and their functionality would be amplified if piezoelectric leaf assemblages prove capable of harvesting significant wind energy. The topic of large-scale vibration energy harvesting, per se, is treated elsewhere [10].

To adapt piezo transducers to plant structures it is necessary to consider frequencies, stress levels, and stochastic movement *vis a vis* modes that effectively excite piezoelectric generators. Piezoelectric elements act as capacitors that self-charge under stress, and capacitive coupling introduces serious challenges to energy harvesting schemes, including impedance mismatch of source and load, dissipation of low frequency signals by RC filtration, and parasitic capacitance. Unique properties of particular materials, e.g., PVDF, electroactive paper, and PZT also set boundary conditions on empirical models.

Here we analyze power conversion by bioinspired plant designs at different frequencies and load resistance, in controlled and natural wind. Our Kynar-based cottonwood mimics improved upon performance of the “vertical flapping stalk” [5], but scaled to the size of a cottonwood tree, power was << daW. Cattail-inspired models with stacked elements showed progressive rise in power with capacitance, but remained << daW per acre. Although our results discourage focus on piezoelectric schemes, alternative methods like the triboelectric system pioneered by Wang’s group [11] may offer a more productive test of wind energy harvesting by botanic mimics.

## Results and Discussion

### Motional frequency and stress

Excitation frequency is a crucial determinant of power output by piezoelectric elements, due to small energy yields per cycle (Figure B in S1 File). Thus it is useful to know the frequency range of plant motions in wind. For quaking aspen (*Populus tremuloides*) the dominant flutter frequency of leaves is ~ 3–5 Hz in wind of 2–4 m/s [9, 12]. Alfalfa stems exhibit an oscillatory decay (~ 1.3 Hz) to neutral position after an initial mechanical displacement [13]. Bending frequencies of alfalfa plants in turbulent wind of 1.1 to 6.9 m/s have been inferred from the power spectra of sun flecks striking under the canopy [14]. A prominent peak occurs at 1–2 Hz in wind speeds of 1.2–6.9 m/s, and at 6.9 m/s the major spectral peak spanned ~ 0.8 to 3 Hz, with some contribution from frequencies up to 10 Hz. Wind stress applied to tree trunks of various species, and the induced trunk excursions, have been measured with strain gauges and high-speed cameras [15]. The power spectral density of trunk sway in *Eucalyptus teretecornus* (red gum) in wind of 17 m/s shows a prominent peak at ~ 0.4 Hz, reflecting a dominant motion at this frequency, and Douglas fir has a strong peak at ~ 0.65 Hz [16]. Upon vigorous agitation by mechanical shakers used at harvest, olive tree frames exhibit normal modes at 20.2 and 37.7 Hz [17]. Short of gale force winds, trunk movements at these frequencies are unlikely to dissipate much wind energy (but see [18]). Thus, wind-driven motion of aboveground plant structures appears concentrated in a band from sub-Hz to a few Hz.

**Fig 1:**
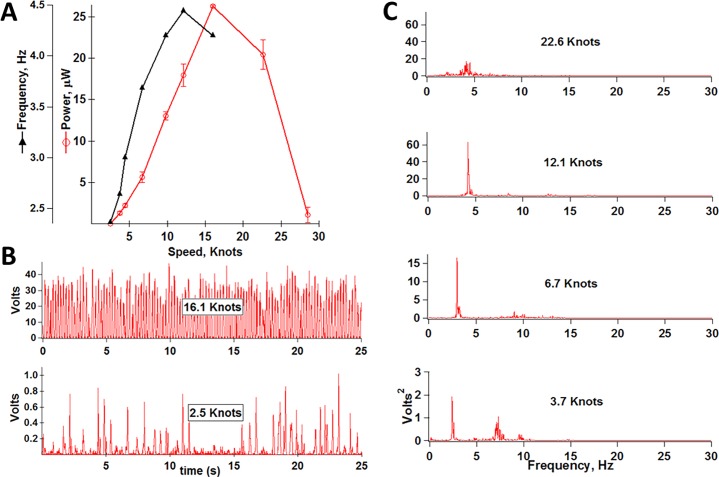
Properties of model cottonwood leaf in fan-generated wind: (A) Power dissipated across 10 MΩ load (circles), and frequency of major band (triangles) as function of wind speed; (B) Raw voltage data at two wind speeds; (C) Power spectra at four wind speeds, showing shift of major band to higher frequencies with increase in wind speed. Leaf was angled 60 degrees downward from horizontal.

The central role of frequency narrows a search for piezo location to small features like leaves (but see Appendix A in S1 File and S1 Mov). As well, non-chaotic is a more effective stimulus than is random motion. We modeled cottonwood leaves because their vertically flattened petioles compel blades to flutter side-to-side, concentrating energy into one oscillation mode. A laminated PVDF strip measuring 6.15 x 1.22 cm serving as petiole was attached to a faux leaf blade made of double thickness lamination plastic. In fan-generated wind of steady velocity this construct mimicked the side-to-side oscillations of live cottonwood leaves (S2 Mov) and had similar threshold wind speed (≤ 3 knots). Fig 1A shows power generated as a function of wind speed, and Fig 1B shows primary data from the rising phase of response. Both frequency and peak voltage increased with wind speed. Flutter frequency measured from slow motion video was ~ half the electrical frequency, as expected with full-wave rectification. Leaf motion became chaotic at ~ 23 knots and nearly ceased at ~28 knots. In the initial, rising phase, power was concentrated in a narrow band that shifted to higher frequencies with wind speed, as indicated in Fig 1C and by the triangles in Fig 1A, reaching ~ 4.5 Hz at 16.1 knots.

**Fig 2:**
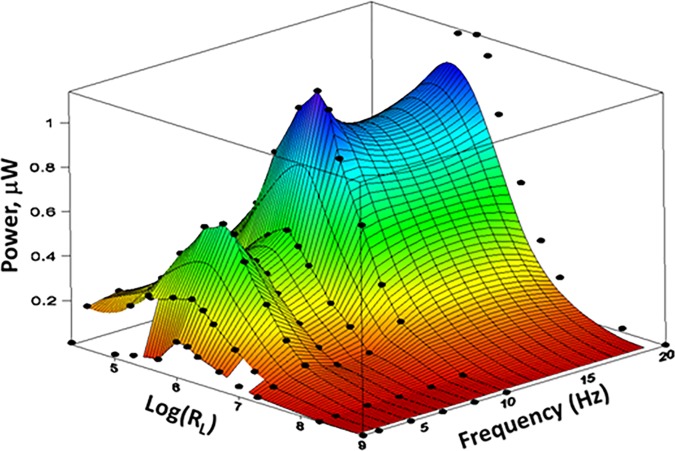
Effect of excitation frequency and load resistance on power output from PVDF strip exposed to repetitive pulses of N_2_ gas at 34 PSI. Optimal R_L_ corresponds to capacitive reactance of piezo. Sweet spot appears to exist at ~ 4 Hz, perhaps due to mechanical resonance.

Fig 2 shows power produced by the same PVDF strip, sans leaf blade, excited by pulses of nitrogen gas at 34 PSI. Pulsatile air excitation allows more precise control of frequency than does steady airflow. For this piezo, power continued to increase with frequency to at least 20 Hz. It jumped disproportionately at 4 Hz, possibly due to mechanical resonance. Greater power output by the complete leaf model likely derives from a larger proof mass (leaf blade), and a bending moment demonstrably less for the partial system. For all frequencies there was an optimal load resistance, also observed in the experiment below.

**Fig 3:**
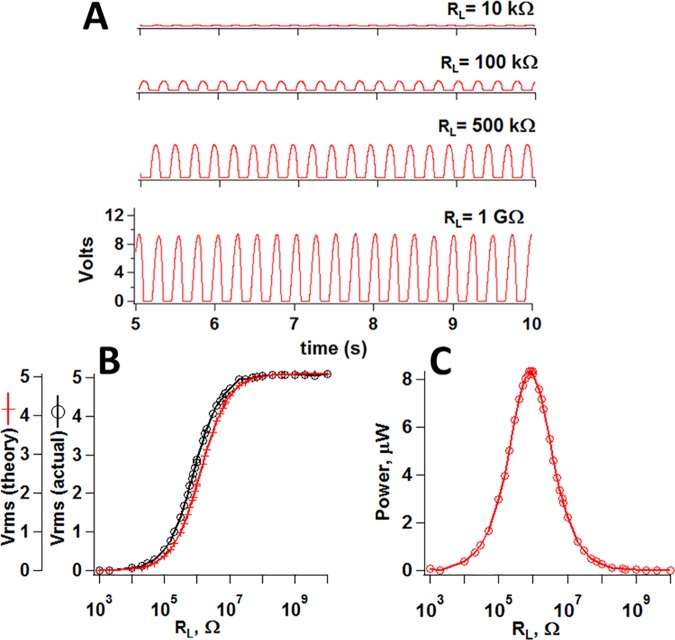
Effect of load resistance on energy conversion by a triplex stack of PVDF elements excited by repetitive pulses of N_2_ gas at 4 Hz: (A) Primary data show progressive drop in voltage with R_L_. (B) Measured (circles) and theoretical (crosses) RMS voltage drops across load resistance; (C) Effect of R_L_ on power (V^2^/R_L_), with optimal harvesting near 1 MΩ.

Fig 3 shows measurements made on a triplex stack of 15.6 x 1.88 cm PVDF strips that was suspended from its base and flexed periodically by pulses of nitrogen gas at ~ 23 PSI. Power rose to a maximum near 4 Hz and decayed sharply above this frequency (not shown). In bending a piezoelectric cantilever, strain (and voltage) is proportional to displacement perpendicular to the film’s #1 axis length (Appendix D in S1 File). At frequencies ≤ few Hz, oak or cottonwood leaf-sized PVDF cantilevers bend and relax a few cm in response to repetitive bursts of gas at ~ 23 PSI. At higher frequencies the response was too sluggish to allow cm-scale displacement and relaxation, though stress from each air burst was greater than the pressure difference (~ 3–30 Pa) across the device due to modest wind (2.2–6.7 m/s).

### Impedance matching

Impedance mismatch of source and load is a huge barrier to wind energy harvesting by piezo-plants. Piezoelectric elements have high output impedance dominated by capacitive reactance, Xc, (Xc = [2π^.^F^.^C]^-1^), where Xc << ohmic resistance of PVDF [volume resistivity 10^14^–10^15^ ohm-cm]. At a frequency (F) = 4 Hz, Xc of a single 28 μm thick PVDF element considered earlier is ~ 4 MΩ (C = 10 nF). Fig 3A shows rectified voltage transients in a triple stack of 15.6 cm x 1.88 cm PVDF strips suspended from their base and excited by repetitive bursts of N_2_ gas (34 PSI) applied to their tip. Note the decrease when R_L_ was reduced from 1 GΩ to 10 kΩ. The load resistance in series with impedance of the piezo forms a voltage divider, and the fraction of source voltage realized across load is R_L_/(R_L_ + Xc). Maximal voltage is observed at R_L_ >> Xc. Fig 3B shows reasonable agreement between measured and theoretical RMS voltage over a broad range of R_L_. Voltage was maximized beyond ~ 100 MΩ. In principle, the optimal efficiency of power transfer to load occurs for R_L_ = Xc. Fig 3C shows that with a PVDF piezo approximately the size of a cottonwood leaf, optimal efficiency of energy transfer to load occurred in the MΩ range, near Xc. A rechargeable Li^+^ ion or NiMH battery has internal resistance < 1 Ω, so for direct charging, transfer efficiency < 2.5 x 10^−5^%.

Harvesting efficiency can be increased by impedance matching through several methods, including buck boost converters and synchronized switch harvesting on inductor (SSHI) or capacitor (SSHC) [19]. Synchronized switch harvesting is effective for periodic signals like those induced by engine vibration, and the stereotyped flutter of *Populus* leaves might be amenable to SSH. But at 20% efficiency—a liberal estimate, maximum power is several orders too small to support production of daW by devices the size of a large tree.

### Charge production

**Fig 4:**
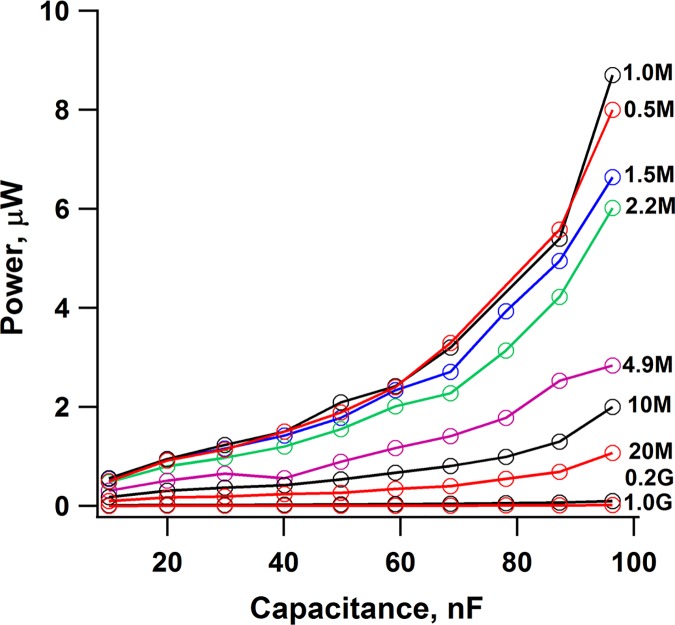
Power generation by a stack of 10 PVDF elements attached to base of 48 cm cattail-inspired model, flexed at 1.3 Hz by a gated air stream (11 knots). Individual elements connected in parallel to increase C step-wise. Approximate ten-fold increase in power with ten-fold increase in C. For R_L_ = 1 MΩ, Fc = 16.7, 2.3 and 1.7 Hz at 1, 7 and 10 elements connected in parallel.

Charge production is a major intrinsic constraint on mechanical-to-electrical conversion by piezo devices. Peak currents can be amplified by increasing the electrode-coated area of a piezo, or by parallel wiring of a stack of mechanically synced elements. However, the benefit of increased area can be offset by parasitic capacitance (Appendix B in S1 File), likewise, stacking reduces compliance. To test if a useful compromise can be met in faux plants we modeled cattails (e.g., *Typha latifolia*) which move somewhat like cantilever beams, more uniformly along their length than does a deciduous oak leaf. Fig 4 shows power produced by a stack of 10 PVDF elements attached to the base of a simplified model comprised of a plastic ruler 19” long and flexed at 1.3 Hz by a gated airstream at 11 knots. Capacitance was increased step-wise by parallel connection of successively greater numbers of elements out of ten in the stack. At R_L_ = 1 MΩ, power increased with capacitance, especially obvious at C ≥ 70 nF, where motional frequency (1.3 Hz) was close to the corner frequency of the RC filter (Appendix C in S1 File). At C = 100 nF, the model leaf produced 9 μW at 1.3 Hz, a higher frequency than living cattails normally experience (direct observation). At ~ 500 *Typha latifolia* plants per acre and an average of 5.5 leaves per plant (S1 Image), power conversion scaled to 0.025 W/acre.

In separate experiments, freshly-harvested cattail leaves were fitted with PVDF strips, and currents recorded across a 10 Ω shunt resistor (Methods). Leaves were bent by airflow from a reciprocating fan (~1 Hz) pushing air at 10 knots, and by manual displacements (~ 2 Hz). Duplex and triplex stacks of 15.6 x 1.9 cm elements were attached directly within the leaf axil or 21.5 cm above it. Figure C in S1 File shows that at neither location nor frequency did stacks generate > 4 μA, although measured across a 1 MΩ load, power was greater for 30.8 than 20 nF (not shown). Interestingly, the duplex attached 21.5 cm above the leaf axil generated RMS current similar to the double stack located in the leaf axil, with components at higher frequencies than for the axil.

To consider the potential performance enhancement of increasing transducer size, envision stacks of 10 PVDF elements described above (15.6 cm x 1.88 cm; C = 10 nF each) but now expanded 25-fold lengthwise and 4-fold in width. The polarization axes are aligned, elements wired in parallel and the stacks are aligned with the long axis of a tree trunk. If each cycle of trunk sway stresses stacks similar to a 10 knot wind driving the 15.6 cm x 1.88 cm element at 4 Hz, a naïve assumption, then at a trunk frequency of 0.5 Hz each stack could generate 1 mW. To generate 80 W would require 80,000 transducers, ignoring inevitable power loss from impedance mismatch or parasitic capacitance. The increased piezo area would lower capacitive reactance to 32 kΩ, still 10^3^ fold greater than internal resistances of batteries (below). The corner frequency of the high pass RC filter in series with a 1 Ω load resistance (5–10 batteries) is ~ 16 kHz, a few thousand times faster than plants move in wind. It might require a Giant Sequoia to accommodate 8 x 10^4^ such transducers, certainly not a convenient backyard plant.

### Tree size projected from capacitor energetics

Piezoelectric transducers behave as capacitors that self-charge under stress. Energy stored across a capacitor is E = ½ CV^2^, with C the capacitance and V the voltage. A 28 μm-thick PVDF sensor 15.6 x 1.88 cm with Ag electrodes (and unilateral 127 μm Mylar backing) has C ~ 10 nF. When subjected to stress similar to that of 10-knot breeze it readily produces open circuit voltage ~ 20 V. At 20 V, energy stored per cycle is ~ 2 μJ, and at a frequency of 4 Hz (natural flutter), max power is ≤ 8 μW. For simplicity this ignores frequency-dependent filtering, and overestimates power. How many such capacitors (leaves) would it take to generate 80 W to 1 kW in a 10 knot wind? Assuming 100% transfer to load and all leaves constantly fluttering—naïve assumptions, 10 M to 125 M synthetic leaves would be required. Apart from cost, the environmental impact would be non-negligible and the spatial footprint in excess of a residential turbine generating equal power. At 10 knots wind power density is ~ 6 W/sq. ft. If leaves sweep an area 5 times greater than their own, efficiency is ~10^−4^%.

### Material constraints

As noted above, the utility of small piezo devices for wind energy harvesting is handicapped by small charge per cycle. The piezoelectric charge coefficient d_3n_ for PZT is 5-fold larger than that of PVDF (1.1 x 10^−10^ vs. 0.23 x 10^−10^ Coulomb/Newton) [20]. This potential advantage is offset by a 21-fold lower voltage coefficient (g_31_), 4.2-fold higher density, and 10–20 fold lower compliance than that of PVDF. It would be futile to construct synthetic leaves 4 times heavier and 10 to 20-fold stiffer than real leaves. Macrofiber composites (MFC) of PZT are less dense and more compliant than is conventional PZT, and in our cattail-inspired model a single MFC (C = 185 nF) produced as much power as did the PVDF stack (98 nF) under the same conditions (Figure D in S1 File). However, stacking of MFC is infeasible beyond ~ 2 or 3 elements, as MFC are less compliant than biological leaves or petioles (see, e.g., [21], [22]). The elastic modulus of oak leaves and water lily petioles is 20–50 MPa, whereas Young’s moduli of PZT MFC are > 15 GPa [23], 10-fold larger than elastic moduli of sunflower petioles [24].

No flexible materials of which we are aware, including cellulose-based EAPap (max d_3n_ 0.07 x 10^−10^ C/N) [25], has d_3n_ values orders greater than that of PVDF or PZT. In one crystal orientation the perovskite Pb(Zn_1/3_Nb_2/3_)O_3_–PbTiO_3_ has a maximal piezoelectric charge coefficient (d_33_) of 2500 pC/N [26], ~ 20- and ~ 100-fold greater, respectively, than those of PZT and PVDF. A 100-fold boost is inadequate to validate piezo-plant schemes. The protein prestin, a piezoelectric actuator of sound amplification in the cochlea, has a maximum sensitivity of 20 μC/N, 10^4^ greater than that of the perovskites [27]. Were a prestin construct expressed in bionic plants, in accessible anatomic sites, it would be a formidable challenge to couple membrane potential changes to charging of batteries.

### Electromechanical coupling factor

The conversion of mechanical to electrical energy by a piezo has a maximum efficiency characterized by k^2^, where k is an electromechanical coupling factor. The magnitude of k varies with excitation mode and material, but for PVDF excited in bending mode, k_31_ = 12%, i.e., PVDF has a theoretical limit of 1.4% for converting mechanical to electrical energy. In theory, induction-based turbines are capable of ~ 70%, and in practice they average 20–30%. PZT and BaTiO_3_ ceramics have k_31_ values ~ 30% and ~ 21%, respectively, corresponding to 9% and 4% conversion efficiencies [20]. This theoretical advantage over PVDF is counterbalanced by other material properties noted above.

### Summation over leaf population, outdoors

**Fig 5:**
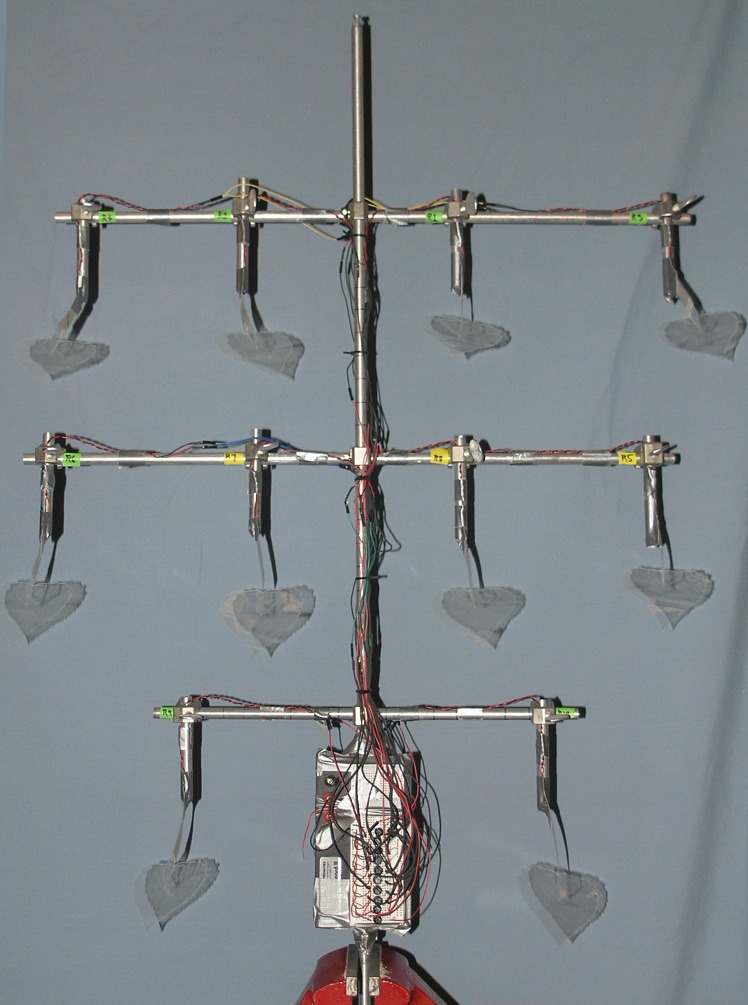
Cottonwood-shaped plastic leaves mounted on aluminum trellis. Output from each Kynar-based petiole was rectified before summation over leaf population.

**Fig 6:**
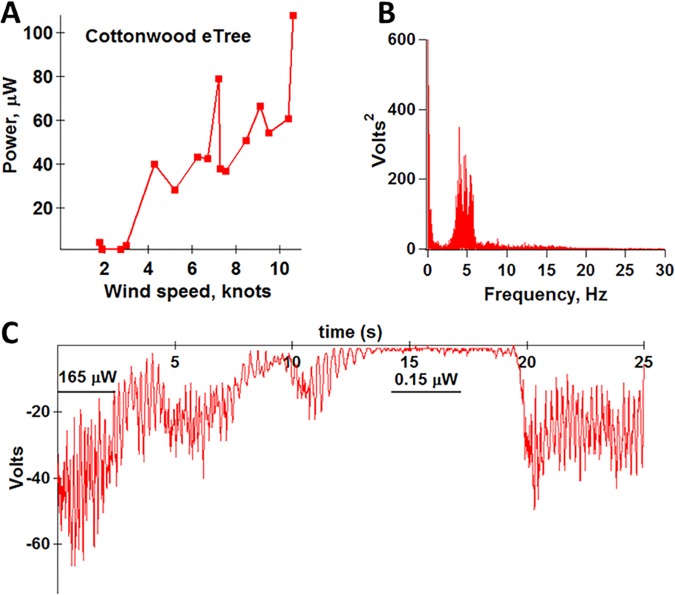
Power output by faux cottonwood trellis as function of wind speed. (A) Average wind speed estimated from mean of highest and lowest speed during the 25 s recording period. At ten knots, power was ~ ten-fold that of single cottonwood mimic indoors. (B) Power spectrum of single leaf during 500 s, in wind fluctuating from 7.5 to 9 knots. (C) Primary voltage data show variation due to fluctuating wind speed and direction.

On a living tree in wind, a sizeable portion of leaves can sit in a wind shadow created by dense foliage on the upwind side. Also, in a stiff breeze many fully-exposed leaves can remain pushed to an extreme, immobile position for long periods (S1 Mov). We asked how power sums over single leaves in our piezoelectric cottonwood model. A primitive piezoelectric tree was fashioned from ten faux cottonwood leaves mounted on a trellis (Fig 5), and power was measured outdoors in natural wind (S4 Mov). Whole-tree power at 10 knots was ~ 10-fold greater than for one synthetic cottonwood leaf indoors at 10 knots (Fig 6 vs. Fig 1). Here, average wind speed was estimated as the mean of highest and lowest speed during the 25 s recording. For this small trellis with well-spaced leaves, its plane perpendicular to wind direction, summation was as predicted for parallel wiring of rectified outputs. As the complexity of the faux tree goes up, and leaves are attached at a density and symmetry more typical of living trees (3d vs 2d), an optimal power is realized with less than 100% of leaves contributing.

### Airflow in wind tunnels vs. outdoors

**Fig 7:**
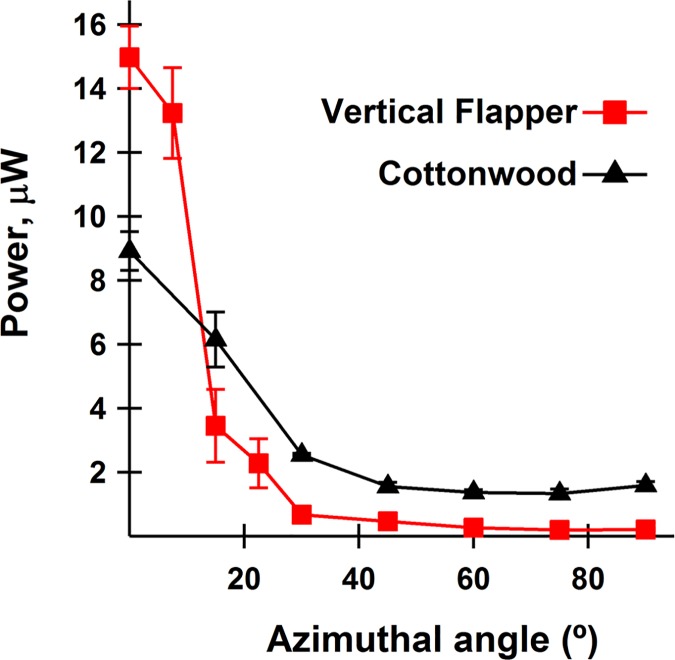
Directional sensitivity of power output from vertical flapping stalk and cottonwood leaf models. Power was maximal for both systems when PVDF petiole was oriented edge-on into wind (8.9 vs. 7.5 knots for flapper vs. cottonwood) and decayed with increase in azimuthal angle. Max power was greater but decayed more steeply for vertical flapping stalk. R_L_ = 10 MΩ both systems.

Previous contributions tested piezoelectric leaves indoors in wind tunnels (3–5, 28). Optimization of a system using laminar wind of constant velocity does not yield a system optimized to function in natural wind. In nature, wind gusts, changes direction, and is rendered turbulent by neighboring leaves and branches within the plant. Fig 6B is a power spectrum of a single cottonwood leaf on the faux tree, in actual wind. It showed a similar flutter frequency to natural cottonwood leaves despite its larger-than-life petiole (S4 Mov). The outsized petiole may help explain why flutter was more sensitive to changes in wind direction than are leaves of authentic cottonwood trees. Our cottonwood model outperformed the vertical flapping stalk (Fig 7) in this regard, but was not as versatile as living cottonwoods; i.e., viable leaves manifest flutter over a broader range of angles of incidence. The nature of leaf attachment to, and mobility of, supporting branches is a crucial determinant of leaf motion in natural wind.

### Selected previous work

Priya’s group constructed tiny windmills coupled to piezoelectric bimorphs that produced 5 mW across a 20 kΩ load in a 10 mph airstream [28]. Windmills convert airflow to periodic motion effective for piezo-based harvesting. To harvest ≥ daW by mimicking often chaotic and low frequency motions of plants is a more complex challenge. A decade ago Rick Dickson inspired discussion of wind energy harvesting by piezo-plants (Dickson, R.M., personal communication). Unfortunately, his approach of “…utilizing large arrays with piezoelectric cells connected in series…” was inherently flawed. Series or parallel multiplexing of piezo elements is possible when elements are excited in-phase, but connecting large arrays of independent elements in series yields zero net voltage.

The British company Solar Botanic patented a “Nanoleaf” that captures sunlight and ostensibly, wind energy [2]. A scaling issue suggests the futility of wind energy harvesting by Nanoleaves: Their “millions and millions of pW,” nowhere actually measured and published, equates with μW per leaf. Allowing an average of 10 μW per leaf and 500,000 leaves—a substantial tree, power from wind energy would be ≤ 5 W.

In an ingenious construct Li et al (2011) fashioned a piezo-leaf that produced “high” power at low frequency [5]. Their vertical flapping stalk is a PVDF petiole hanging vertically and attached to a triangular plastic blade, the planes of piezo and leaf blade parallel to each other and perpendicular to ground. Under ideal conditions, i.e., with the PVDF strip oriented perfectly edge-on into wind, downstream of a bluff body, it produced more power (100–200 μW across 10 MΩ) than our cottonwood mimic. We modeled their system from available information, and sans bluff body it produced 15–100 μW at wind speed of 8–18 knots (Fig 7 and S3 Mov). Video records showed high amplitude flexion and torsion, which likely explain high power but also distressing noise (65–80 dB at 10 knots).

We asked how output under ideal conditions compared to that at different azimuthal and altitudinal angles, as present in wind gusts outdoors. The faux cottonwood showed striking dependence on altitudinal angle. In 8 knot wind it generated 0.2, 22, 8.5 and 9.9 μW at angles of 0, -30, -60 and -90° from horizontal. Although not addressed by Li et al., we found that the flapping stalk stopped flapping for any but a small range of azimuthal angles nearly parallel to wind direction (Fig 7). Integration over 90 degrees gave 273 vs. 221 μW-degree for cottonwood vs. vertical flapper. Although the flapper beat the cottonwood in max power, the cottonwood capitalized on changes in wind direction better than did the flapping stalk.

A bluff body boosted power from the flapping stalk 10-fold under idealized wind tunnel conditions [5]. Bluff bodies also were used in wind energy harvesting by a simpler system yielding sub-μW power [29]. Clearly, a static bluff body placed upstream is of no value when wind shifts direction, blowing from leaf towards bluff body. Fig 6C shows output from a trellised cottonwood, outdoors in natural wind (S4 Mov). During 25 s, as wind changed direction and speed, power ranged from 0.15 to 165 μW. We conclude that modest changes in wind direction, and turbulence caused by nearby leaves and branches, compromise performance of e-Trees in ways not considered in previous work. Further, to employ the vertical flapping stalk or other piezo-leaves in real-world settings would require large collections of leaves. Yet to assemble >10,000 flapping stalks or cottonwood mimics, each leaf occasionally producing 100 μW on a windy day, would yield ≤ 1 W. By comparison, a solar panel having much smaller footprint can average 200 W on a sunny day. An alternate mechanism of energy transduction, or electroactive materials of orders greater energy density than present ferroelectrics, may place the piezo-plant idea on firmer ground.

### Conclusions

Our findings indicate that generation of ≥ daW from piezoelectric botanic mimics of practicable size is not a near-term reality. Transduction schemes of greater energy density such as triboelectric conversion [11] or perhaps electrostriction of flexible polymers [30] may be worthy of testing in synthetic plants. Although valid concerns attend displacement of living with artificial plants, to demonstrate mid-range power for off-grid use in a modest-sized plant mimic would provide strong impetus for real world testing and possible development of such devices.

## Materials and Methods

Several types of PVDF sensors were donated by or purchased from Measurement Specialties, Inc. (Hampton, VA). Macrofiber composites of PZT (d31-MFC) were purchased from Smart Material Corporation (Sarasota, FL). Resistors, capacitors, diode bridge rectifiers, op amps and other electronic components were from Newark (Gaffney, SC) or Digi-Key (Thief River Falls, MN). Capacitance was measured with a BK Precision 890B capacitance sorter. Airspeed was measured with a hand-held anemometer (BK Precision #731A).

In some experiments (Fig 3) voltage measurements were made with a USB digital scope and stimulator (Stingray DS1M12) from EasySYNC Ltd. (Hillsboro, OR). Digitized data from the Stingray interface were exported from EasySYNC into Excel. In most experiments a Tektronix digital storage scope (TDS 2002C) was used with Open Choice Desktop, and data exported to Excel. IGOR Pro (Wavemetrics, Lake Oswego, OR) was used to analyze and plot all data.

Piezo output was rectified with diode bridge rectifiers (Multicomp AM154 and Multicomp W01), the signal passed through variable load resistance, and RMS voltage across R_L_ (Figure A in S1 File) measured over periods of 25 s to 9 m. Power was estimated as [Vrms]^2^/R_L_. In living cattail experiments, currents were measured from the voltage drop across a 10 ohm precision resistor, using an LTC 1050 op amp with feedback resistance such that gain = 10^4^. A capacitor of similar capacitance to the piezo was used in a few trials to smooth the signal. For a cattail mimic, ten DT4 elements (MSI part # 1-10022149-0) were attached back-to-back with their electrical axes aligned and electrodes wired in parallel with 3M™ EMI Shielding Tape #1183. Each piezo was addressable individually. 3M VHB adhesive transfer tape (F9460 PC) was used to bond elements to each other and to a 12 inch plastic ruler (Wescott B-70) extended to 19” with beavertail sail. DT4 elements are ~ 40 μm thick (PVDF = 28 μm) and VHB tape ~ 50.8 μm. Cattails are not perfect cantilevers, as they often are twisted ≥ one revolution lengthwise, and their cross sectional shape and area vary lengthwise, the distal ~ ¼ of leaf more flexible and strained by wind than the proximal 1/3. However, to capitalize on this tip geometry is difficult because piezo stacks here limit strain. The number density of cattail plants in a local stand (S1 Image) was measured by counting individuals in two 100 sq. ft. domains.

A rotary fan at constant speed and wind direction and for higher speeds a heavy duty dust collection system (Penn State Industries, model DC-3XL) were used to induce flutter of cottonwood mimics. In some experiments a pneumatic PicoPump (World Precision Instruments, Sarasota, FL) was used to deliver repetitive bursts of nitrogen gas at 23 or 34 PSI to the tips of elements anchored at their base. Gating frequency was controlled by TTL output from the USB scope. Artificial cottonwood leaf blades were fashioned from double-layered lamination plastic or 160 μm Tyvek^TM^ on a laser cutter programmed with authentic cottonwood leaf template. Of several materials tested, Tyvek and lamination plastic allowed the most consistent side-to-side motion of the PVDF petiole at low wind speeds (S2 Mov), mimicking the flutter of natural cottonwood leaves. The petiole was constructed from a laminated PVDF element (MSI #LDT2-028K/L). An earlier version with larger blades (13 x 14 cm), and a 15.6 x 1.88 cm piezo-petiole produced much less power than the smaller device.

A synthetic cottonwood tree was made from an aluminum trellis and plastic leaves anchored to the branches with duct tape (S4 Mov and Fig 5). Leaves were aligned at 0° (azimuthal) to wind direction, and ~ 45° down from horizontal. Rectified output from each leaf was connected to a parallel bus and voltage measured across 10 MΩ load.

## Supporting Information

S1 File Appendices and supplementary figures. (DOCX)

S1 Image Cattail stand used to estimate # density. (GIF)

S1 Mov Maple tree, ~ 15 knot wind. (MOV)

S2 Mov Cottonwood leaf mimic, SloMo. (MP4)

S3 Mov Vertical Flapping Stalk, SloMo. (MP4)

S4 Mov Cottonwood trellis, McFarland Park (MP4)
