# Supplementary material for: Wind Energy Conversion by Plant-Inspired Designs
Source: PLoS One. 2017 Jan 13;12(1):e0170022. doi: 10.1371/journal.pone.0170022 (PMC5234829; doi:10.1371/journal.pone.0170022)

Supporting Information File 1

Appendix A. Assume a leaf weighs 0.5 gram, it has a regular flutter at 4 Hz, and maximal displacement is ~ 5 cm per cycle. Full-grown cottonwood leaves were measured and found to weigh 270 to 320 mg. Their widest dimension was ~ 8 cm, and related poplar leaf flutter is ~ 4 Hz in 12 mph wind. At 4 Hz, the mass travels at an average speed of 5 cm/cycle x 4 cycles/s = 20 cm/s. During each cycle it accelerates twice from 0 to 20 cm/s over a period of 125 ms, so average acceleration is (20 cm/s)/0.125 s ~ 160 cm/s**2. It reaches a greater value greater in each cycle, and it decelerates to zero velocity at the extrema. The force F = m*a = 0.5 gm * 160 cm/s**2 = 80 gm*cm/s**2 = 80 dynes. Energy imparted per cycle is F*d = 80 dynes * 5 cm = 400 ergs = .04 mJ. At 4 Hz, the power is .04mJ/cycle * 4 cycles/s = .16 mJ/s, or 160 μW. The motion of 500,000 leaves can thus dissipate ~ 80 W of wind energy. This says nothing about the fraction of incident wind energy that is converted to heat, or to leaf and branch sway. Although substantial energy appears dissipated by branch movement, curvature in general is too gradual to stress appreciably a piezo short enough to avoid internal loss from parasitic capacitance.

Figure A. (Below) Schematic of circuitry. Output from each piezo-leaf was full-wave rectified before parallel multiplexing of multiple sources. Load capacitance (C_L_) was added in selected experiments.

**
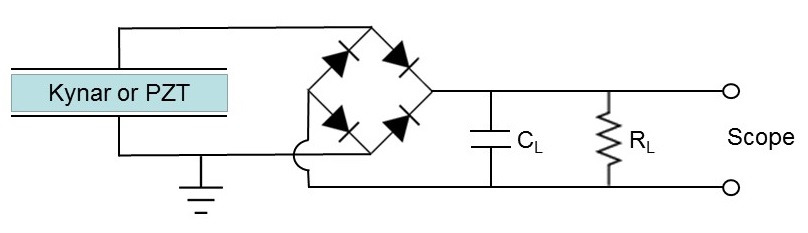
**

Figure B. (Below) Time-dependent voltage signals generated by a 110 μm thick PVDF element that was bent, maintained in the bent conformation, and then allowed to relax to its original shape after 2h. Transients are due to charge swapping between the plates of the capacitor and external circuit, and they occur only during initial film displacement and during relaxation. A major constraint of piezoelectric energy harvesting via biomimetic plant designs is that even though stress can be ~ constant for periods of min to h, charge flow is restricted to periods of change in stress, which means changes in wind direction, speed, or anatomic designs prone to cycle in steady wind. Plastic deformation and creep thwart comparison of total charge for ON and OFF currents. Still, integrated areas for relevant currents were approximately equal.

**
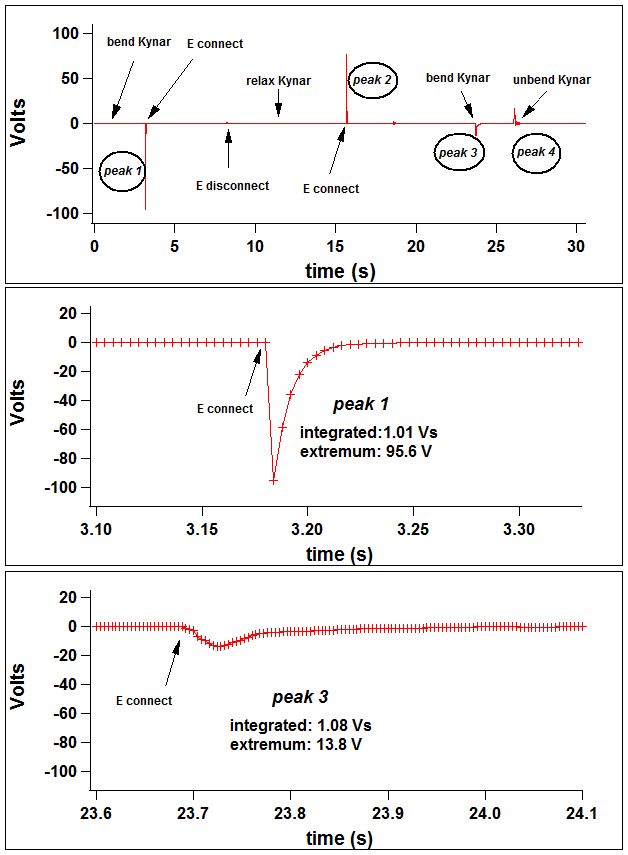
**

Appendix B. The charge, Q, produced across a piezo increases with area, A, of electrode-coated transducer: Q = d_3n_X_n_A (n = 1, 2 or 3), where Xn is the stress (N/m^2^) applied in the relevant direction, and d_3n_ are the piezoelectric charge (strain) coefficients in each of the three axes, 3 being the polarization (thickness) axis. For a given stress, charge production can be increased by increasing the electrode-coated area of an element, or by parallel wiring within a stack of elements moving in-phase. These benefits are offset by reduction of compliance in stacks and by parasitic capacitance in large transducers. By capacitance charging, regions under low stress parasitize energy from those under high stress, and net voltage is reduced by adjacent areas of opposite curvature.

Figure C. (Below) Currents induced by motion of freshly harvested cattail leaves fitted with triple (A) or double (B, C) stacks of 6.13” x 0.74” PVDF strips (28 μm). Leaves flexed through a 4-foot span (linear tip-to-tip distance) by a reciprocating fan or by hand.  Stacks anchored directly within leaf axil (A and B) or beginning 21.5 cm above it (C).


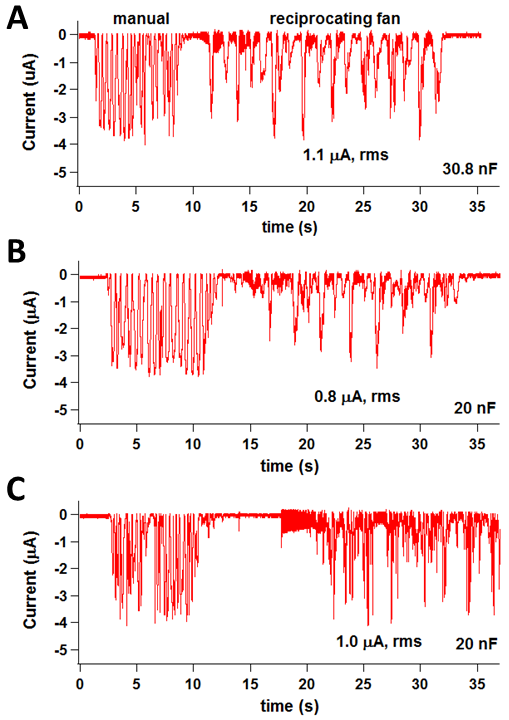


Appendix C. RC Filtration. Kynar film elements have an intrinsic capacitance (380 pF cm^-2^ for 28 μm thickness), which acts in series with load resistance to provide a high-pass filter for the electrical output. The cutoff frequency Fc = (2πR_L_C)^-1^ so with a 1 MΩ load and 28 μm strips having C = 2.8 nF (LDT2/K element), Fc ~ 58 Hz. Well above 58 Hz the voltage drop across a load is equal to open circuit voltage and directly proportional to stress, but as frequencies drop below 58 Hz less power is transferred to load, and output is proportional to the rate of change of applied stress. 58 Hz far exceeds natural frequencies of wind-driven motion of living plants. One can lower the cutoff frequency by increasing capacitance, i.e., increasing the film area: C = ε^.^A/t, where ε = permittivity, A = area of electrode-coated film, and t = film thickness. There is a tradeoff between frequency and capacitance. Increase in either lowers capacitive reactance, but stacking reduces strain by increasing flexural rigidity, and anatomic structures conducive to stack insertion inherently move at low frequencies.

Appendix D. Open circuit voltage increases linearly with stress, with piezoelectric thickness, t, and it is invariant with the area of electrode-coated transducer:

V = g_3n_X_n_t (n = 1, 2 or 3).

X*n* is stress applied along axis *n*, and g_3n_ are the relevant voltage (stress) coefficients.

Figure D. (Below) Power output from one MFC (C = 185 nF) mounted on same cattail-inspired leaf as in Fig. 4. Wind at 6.5 knots gated at 1.3 Hz.


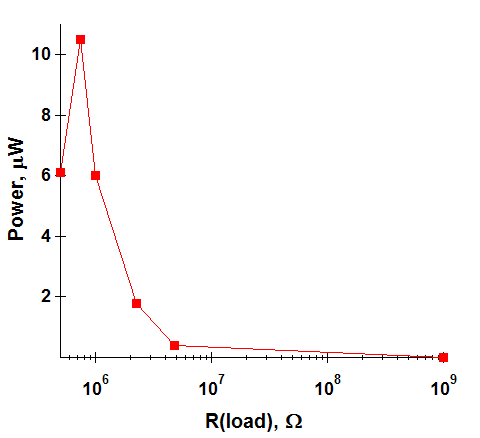

Supplement: S1 File — (DOCX) [file pone.0170022.s001.docx]
