# Supplementary figures and images for: Wind Energy Conversion by Plant-Inspired Designs
Source: PLoS One. 2017 Jan 13;12(1):e0170022. doi: 10.1371/journal.pone.0170022 (PMC5234829; doi:10.1371/journal.pone.0170022)

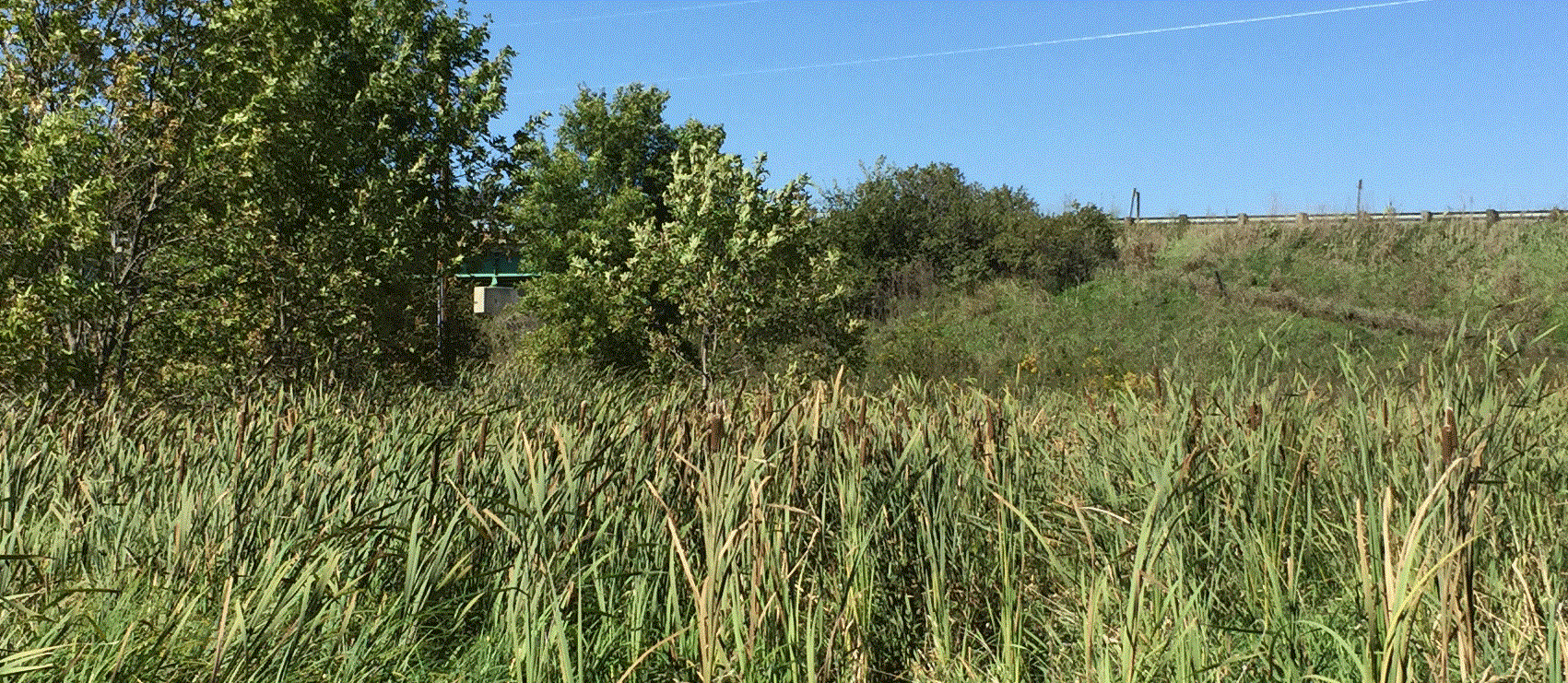

Supplement: S1 Image — (GIF) [file pone.0170022.s002.gif]
